# Primary progressive aphasia: a dementia of the language network

**DOI:** 10.1590/S1980-57642013DN70100002

**Published:** 2013

**Authors:** Marsel Mesulam

**Affiliations:** MD, Cognitive Neurology and Alzheimer's Disease Center, Northwestern University Feinberg School of Medicine, Chicago, IL, USA.

**Keywords:** dementia, language, network, frontotemporal, progranulin, tau

## Abstract

Primary progressive aphasia (PPA) is a clinical syndrome diagnosed when three core criteria are met. First, there should be a language impairment (i.e., aphasia) that interferes with the usage or comprehension of words. Second, the neurological work-up should determine that the disease is neurodegenerative, and therefore progressive. Third, the aphasia should arise in relative isolation, without equivalent deficits of comportment or episodic memory. The language impairment can be fluent or non-fluent and may or may not interfere with word comprehension. Memory for recent events is preserved although memory scores obtained in verbally mediated tests may be abnormal. Minor changes in personality and behavior may be present but are not the leading factors that bring the patient to medical attention or that limit daily living activities. This distinctive clinical pattern is most conspicuous in the initial stages of the disease, and reflects a relatively selective atrophy of the language network, usually located in the left hemisphere. There are different clinical variants of PPA, each with a characteristic pattern of atrophy. The underlying neuropathological diseases are heterogeneous and can include Alzheimer's disease as well as frontotemporal lobar degeneration. The clinician's task is to recognize PPA and differentiate it from other neurodegenerative phenotypes, use biomarkers to surmise the nature of the underlying neuropathology, and institute the most fitting multimodal interventions.

## Abstract (pt)

A afasia progressiva primária (APP) é uma síndrome clínica diagnosticada quando três critérios centrais são preenchidos. Inicialmente deve haver comprometimento da linguagem (afasia) que interfere com o uso ou a compreensão das palavras. Em segundo lugar, os métodos diagnósticos devem determinar que a doença é neurodegenerativa e, portanto, progressiva. Por último, a afasia deve emergir de forma relativamente isolada, sem alterações equivalentes de comportamento ou memória episódica. O comprometimento da linguagem pode ser de tipo fluente ou não fluente, e pode ou não interferir com a compreensão de palavras. A memória para eventos recentes está preservada, embora escores de memória em testes verbais possam ser anormais. Alterações discretas de personalidade e comportamento podem estar presentes, mas não são os fatores que levam o paciente ao atendimento médico ou que o limitam em suas atividades de vida diária. Este padrão distinto é mais evidente nas fases iniciais da doença e reflete a atrofia relativamente seletiva da rede de linguagem, geralmente localizada no hemisfério cerebral esquerdo. Há diferentes variantes clínicas da APP, cada uma com padrão de atrofia característico. Os substratos neuropatológicos são heterogêneos e podem incluir a doença de Alzheimer e a degeneração lobar frontotemporal. A tarefa do clínico é reconhecer a APP e diferenciá-la de outros fenótipos neurodegenerativos, utilizar biomarcadores para inferir a natureza da neuropatologia subjacente e instituir as intervenções multimodais cabíveis.

## INTRODUCTION

Progressive aphasias have been recognized for more than 100 years as illustrated by the case reports of Pick, Sérieux, Dejerine, Franceschi, and Rosenfeld.^1-5^ In many such patients, the aphasia progresses in tandem with equally salient impairments of memory or behavior. In others, as exemplified by the patient initially reported by Sérieux and subsequently examined at autopsy by Dejerine, the aphasia arises as the only salient feature and leads to the distinctive syndrome of primary progressive aphasia (PPA).

The current interest in this syndrome can be traced to a 1982 report of six patients with the relatively isolated emergence of a slowly progressive language disorder.^6,7^ As additional patients became identified throughout the world, the term 'Primary Progressive Aphasia' was coined and diagnostic criteria were delineated.^8-11^ The PPA diagnosis is now made when three core criteria are met: [1] there is a progressive aphasic disorder of recent onset as manifested by gradually intensifying distortions of word usage or comprehension that could not be attributed to more elementary motor or perceptual deficits; [2] the language impairment constitutes the most salient neurobehavioral deficit and the chief impediment to the pursuit of customary daily living activities during the initial stages of the illness; and, [3] diagnostic investigations lead to the conclusion that the underlying disease is neurodegenerative.

In some patients, the principal signs and symptoms are confined to the area of language for as many as 10-14 years. In others, impairments in other cognitive functions can emerge after the initial few years, but the language dysfunction tends to remain the most salient feature and to deteriorate the most rapidly throughout the course of disease.^12^ PPA is a form of dementia since it causes gradual cognitive decline to the point where daily living functions become compromised. It is also an unusual dementia since episodic memory functions remain largely preserved for many years. In contrast to patients with amnestic dementias of the Alzheimertype (DAT), who tend to lose interest in recreational and social activities, some patients with PPA maintain and even intensify involvement in complex hobbies such as gardening, carpentry, sculpting and painting.

Primary progressive aphasia should be differentiated from states of pure progressive dysarthria, speech apraxia, or phonological disintegration where the formation, rather than usage, of words becomes disrupted.^13,14^ It should also be differentiated from DAT and behavioral variant frontotemporal dementia (bvFTD) where word-finding disturbances or a paucity of speech may arise, but on a background of more salient impairments of memory (in DAT) and behavior (in bvFTD).

Age of onset has ranged from the 40s to 80s. However, the majority of patients have had onset before the age of 65.^15^ *Dysarthria* can occasionally arise and contribute to loss of fluency. *Ideomotor apraxia*, sometimes in the form of "sympathetic dyspraxia" in the left hand can be encountered. A more frequent occurrence is the presence of an isolated buccofacial apraxia so that the command to "cough" cannot be followed even though the patient understands the instructions and can perform the action spontaneously when the need arises. *Dyscalculia* is very common, reflecting the anatomical proximity of the brain areas necessary for language and calculations. In some patients, the dyscalculia emerges early and becomes as prominent as any other of the aphasic impairments. Occasionally, all components of the Gerstmann syndrome can be present. A careful neurological examination can reveal subtle *asymmetrical pyramidal or extrapyramidal signs* on the right side of the body reflecting the dysfunction of the language-dominant (left) hemisphere. These signs include mild flattening of the nasolabial fold, widening of the palpebral fissure, asymmetrical posturing of the hand while walking on the heels or edge of the feet, and mild cogwheeling rigidity induced when the other hand is engaged in repetitive tapping movements.

An abrupt onset of the aphasia excludes the diagnosis of PPA. Additional exclusionary criteria include the *early salience* of motor deficits, amnesia, abnormal comportment, associative agnosia, or visuospatial disorientation. Patients with these features may have the phenotypes of motor neuron disease (MND), corticobasal degeneration (CBD), progressive supranuclear palsy (PSP), DAT, bvFTD, or the syndrome of posterior cortical atrophy (PCA), each of which can be accompanied by a non-primary but progressive aphasia. The mere presence of an aphasia is thus not sufficient for the diagnosis of PPA. Brain imaging is part of the diagnostic work-up since any finding other than atrophy that can account for the aphasia (such as neoplasm or ischemic lesions) rules out the diagnosis of PPA.

Additional cognitive, behavioral and motor deficits that independently influence daily living activities arise in the middle or late stages of the disease.^16,17^ We have used the descriptive term "PPA-plus" (PPA+) to designate the fact that the patient had initially fulfilled the diagnostic criteria for PPA but that the aphasia is no longer the only major deficit.^18^ Personality changes (inappropriate familiarity, impaired problem solving, blunted judgment) or asymmetrical extrapyramidal deficits may emerge quite commonly as the disease progresses and reflect the close anatomical association of PPA-causing diseases with those causing bvFTD and CBD.

Diagnosing PPA is easiest when the patient is examined early so that core criteria can be fulfilled explicitly. Occasionally, the clinician will see a patient at a more advanced clinical stage, at a time when the selectivity of aphasia may no longer be ascertainable because of language comprehension deficits or because deficits in other domains have emerged. In such cases, a structured interview with informants can be used to establish whether the aphasia had in fact emerged in relative isolation. A retrospective diagnosis of "possible PPA" is made if such an interview confirms that the diagnostic criteria had been met during an earlier phases of the disease in a patient who now has other deficits as well.

## CLASSIFICATION AND TERMINOLOGY IN PPA

The delineation of classic aphasia syndromes was based on patients with cerebrovascular lesions. Clinical features in PPA seldom fit these patterns, perhaps because the indolent pace of neurodegeneration allows a considerable rewiring of language-related circuitry. The syndromic nomenclature of stroke aphasia has therefore not been all that helpful in the characterization of PPA subtypes. Instead, three cardinal aphasic patterns have been identified in PPA and designated *agrammatic, semantic* and *logopenic* Elaborate guidelines for this classification system were published in 2011.^19^

The agrammatic subtype (PPA-G) is characterized by impairments of grammar (syntax and morphology) but not of word comprehension; the semantic subtype (PPA-S) by impairments of word comprehension but not of grammar; and the logopenic subtype (PPA-L) by intermittent word-finding hesitations without impairments of comprehension or grammar. Fluency, measured in words per minute, is consistently low in PPAG, may be unremarkable or excessive in PPA-S, and is highly variable in PPA-L. Repetition may be impaired in both PPA-G and PPA-L, but not PPA-S. In some patients grammar and comprehension are jointly impaired early in the disease. These patients can be said to have a fourth or 'mixed' subtype of PPA (PPA-M).^15^

The term 'logopenic' was introduced in 1992 to designate patients with preserved grammar and comprehension who had frequent word-finding hesitations and anomia.^9^ The 2011 guidelines introduced the additional criterion of abnormal repetition as a requirement for the diagnosis of PPA-L.^19,20^ According to this more restrictive approach, patients who have impairments of word-finding but not of grammar, repetition or comprehension, remain in limbo because they fail to fit any of the three subtypes. This predicament becomes particularly challenging in the early and mild stages of PPA.^15^ Some of these patients may be said to manifest an 'anomic' subtype of PPA (PPA-A) and may eventually display features of PPA-L or PPA-S.^21^ The PPA-G and PPA-L variants collectively account for what is also known as progressive nonfluent aphasia (PNFA), while the PPA-S variant designates the predominantly aphasic form of semantic dementia (SD).

Specific tests and quantitative parameters have been delineated for the classification of PPA patients according to the 2011 guidelines.^15^ However, the process is quite burdensome and introduces considerable challenges in the differentiation of PPA-G from PPAL. Table 1 offers a simpler descriptive set of guidelines that should be adequate for use in most clinical and even research settings. It does, however, contain a major deviation from the 2011 guidelines. While the 2011 guidelines specify that either agrammatism or effortful/ apraxic speech is sufficient for the diagnosis of 'nonfluent/ agrammatic' PPA, we prefer to differentiate grammar from speech so that agrammatism, by itself, and in the absence of word comprehension impairment, is the critical feature leading to the diagnosis of 'agrammatic' PPA (PPA-G). This departure from the 2011 criteria is in part motivated by the fact that patients with isolated speech and fluency impairments would not fulfill the root diagnosis of PPA.

**Table 1:** Descriptive and simplified criteria for classifying primary progressive aphasia.

**Diagnostic criteria for PPA**	The following three conditions must all be present. 1. A new and progressive language disorder (aphasia) as documented by neuropsychologically determined abnormalities in one or more of the following domains: grammaticality of sentence production, word retrieval in speech, object naming, word and sentence comprehension, spelling, reading, repetition. Isolated impairments of articulation do not qualify. 2. Initial and relative preservation of episodic memory, executive functions, visuospatial skills and comportment as documented by history, medical records and/or neuropsychological testing. 3. Imaging and other pertinent neurodiagnostic test results that rule out causes other than neurodegeneration.
**Agrammatic Subtype (PPA-G)**	Impaired grammatical structure of spoken or written language in the absence of significant word comprehension impairments. Output is usually of low fluency but does not have to be dysarthric or apraxic.
**Semantic Subtype (PPA-S)**	Impaired word comprehension in the absence of significant impairment of grammar. Object naming is severely impaired. Output is motorically fluent but contains word finding hesitations, paraphasias and circumlocutions.
**Logopenic Subtype (PPA-L) **	No significant grammar or word comprehension impairment. Speech contains many word-finding hesitations and phonemic paraphasias. Object naming may be impaired and may constitute the only significant finding in the neuropsychological examination. Current classification systems require repetition impairments for diagnosing this subtype (19).
**Anomic Subtype (PPA-A)**	All features as in PPA-L except that repetition is intact.
**Mixed Subtype (PPA-M)**	Impaired grammatical structure and word comprehension, even at the early stages of disease.

## NEUROPSYCHOLOGICAL CHARACTERIZATION

Standardized neuropsychological tests are helpful for reaching an early diagnosis. However, a strict reliance on neuropsychological tests, most of which depend on verbal instructions, verbal responses, or covert verbal reasoning, may occasionally lead to the erroneous conclusion that areas other than language are also impaired. Scores on the Mini Mental Status Examination (MMSE),^22^ for example, can exaggerate the degree of disability.^23^ Although the language disorder in primary progressive aphasia may interfere with the ability to memorize word lists or solve reasoning tasks, the patient typically has no difficulty recalling daily events or behaving with sound judgment, indicating that explicit memory, reasoning and social skills remain relatively intact.

The neuropsychological examination of the patient with suspected PPA aims to demonstrate the aphasia, characterize its subtype, and identify non language cognitive domains that are relatively spared. Language function can be tested with one of the several clinical batteries designed for this purpose. The Western Aphasia Battery (WAB-R) includes subtests that measure spontaneous speech, word and sentence comprehension, naming, reading and writing.^24^ An Aphasia Quotient, derived from the WAB-R, provides a measure of aphasia severity that can be tracked over time. The grammaticality of sentence production can be tested with the Northwestern Anagram Test (NAT), a measure of sentence construction that does not place demands on working memory or speech output.^26^ Additional information on grammaticality can be obtained with the Sentence Production and Priming Test (SPPT) of the Northwestern Assessment of Verbs and Sentences (NAVS).^27^ Repetition performance can be measured with the WAB. We previously used performance on the 6 most difficult items of tthe WAB repetition subtest to classify PPA subtypes at early and mild stages of impairment.^15^ Surface dyslexia and dysgraphia, very common in PPA-S, can be assessed with exception words from the Psycholinguistic Assessment of Language Processing in Aphasia (PALPA).^28^

Word association and comprehension can be tested with a subset of 36 moderately difficult items (157-192) of the Peabody Picture Vocabulary test, PPVT-IV.^29^ Performance on this particular set of items had been used to construct a quantitative template for subtyping PPA and has become a core component of our testing battery.^25^ Each item requires the patient to match an auditory word representing an object, action or attribute to one of 4 picture choices. Although the PPVT-IV is a word-picture matching task, less than half of the items represent concrete objects. The majority of the remaining words (e.g., salutation, perplexed, culinary) require extensive associative interpretation (i.e., comprehension) of the words in order to match them to pictorial representations of the corresponding concept. Additional assessment of word comprehension can be obtained with tests of word definition and associations.^30^ The Boston Naming Test (BNT) provides a standardized measure of object naming.^31^

Non-verbal faculties should be tested with instruments that minimize interference from the aphasia. Non-verbal knowledge of objects can be tested with the three-picture form of the Pyramids and Palm Trees test.^32^ This relatively simple test should be supplemented with more specialized tests of object knowledge as well as with a standardized questionnaire assessing the patient's use of objects in naturalistic settings.^30^ Episodic memory can be tested with the Three Words Three Shapes (3W3S) test, a measure we previously designed to differentiate DAT from healthy cognitive aging.^33^ This test showed that PPA patients have a selective retrieval impairment for words but not for shapes. PPA patients are therefore likely to forget words they hear or read but not events they experience. The relative preservation of reasoning skills in PPA can be documented with the Visual Verbal Test, a non verbal test of cognitive flexibility.^34^ Visuoperceptual functions can be tested with Judgment of Line Orientation.^35^ Behavioral changes, salient in early stages of bvFTD, but not typically apparent until later stages of illness in PPA , can be assessed with the Frontal Behavior Inventory.^36^

## FUNCTIONAL AND STRUCTURAL NEUROANATOMY

The single most distinctive feature of PPA is the asymmetric atrophy of the language dominant (usually left) hemisphere. Quantitative morphometry shows that the PPA-G subtype is most closely associated with atrophy in the posterior frontal lobe, including Broca's area; the PPA-S subtype with atrophy in the anterior temporal components of the language network, including the temporal pole; and the PPA-L subtype with atrophy in the temporo-parietal component of the language network.^15,17,25,37^ There can be substantial overlap between the atrophy patterns of PPA-G and PPA-L.

Abnormalities of blood flow and metabolism may emerge prior to the detectable atrophy. SPECT or PET may therefore provide more sensitive diagnostic information than structural MRI or CT scans. However, even metabolic imaging may be uninformative during the first several years of disease and the diagnosis may need to be based on the clinical examination alone.^15^ Functional imaging helps to explore the physiological bases of the language impairment. When asked to identify homonyms or synonyms in the course of functional MRI experiments, PPA patients and age-matched controls activate the same components of the language network, including Broca's and Wernicke's areas.^38^ However, the functional connectivity between these two major nodes of the language network becomes disrupted.^39^ It appears, therefore, that abnormal language function in PPA may initially reflect an impairment of information transfer within the language network rather than a failure of activation at network hubs. In comparison to neurologically intact subjects, the PPA patients also display additional aberrant activations within regions of the brain outside of the classic language network.^38^ It is not yet clear whether these aberrant activations reflect compensatory processes or abnormal disinhibition. The latter possibility is supported by the fact that the intensity of the aberrant activations is inversely correlated with performance on a naming test.^38^

## NEUROPATHOLOGY

Post-mortem examinations show that the vast majority of PPA patients have the pathology of either frontotemporal lobar degeneration (FTLD) or of AD. Both major classes of FTLD, one with tauopathy (FTLD-TAU) and the other with TDP-43 precipitates (FTLD-TDP), have been reported.^40^ In the majority of sporadic PPA-G the neuropathology is of the FTLD-TAU type. In the majority of PPA-S, the neuropathology is of the FTLD-TDP type. The remaining 20-30% of patients in these two variants show the neuropathology of AD.^40,41^ In PPA-L, more than half of the cases have AD pathology and the rest FTLD.^40,42^ Quantitative analyses of post-mortem cases showed that PPA patients with AD pathology had higher neocortical-to-entorhinal and left-to-right ratios of neurofibrillary tangles than patients who had the typical combination of AD pathology with an amnestic (rather than aphasic) dementia.^43^ This atypical distribution of neurofibrillary degeneration is consistent with the anatomy of the clinical phenotype in PPA.

Determining whether an individual PPA patient has AD versus FTLD pathology is always challenging. ApoE genotyping or F18-DG metabolic scans do not help in this differentiation. In fact, the ε4 allele of ApoE, which is a major risk factor for Alzheimer pathology in amnestic dementias, is not a risk factor for the type of Alzheimer pathology that causes PPA.^43,44^ Amyloid imaging with PET and cerebrospinal fluid evaluations for phosphotau and beta amyloid may be helpful for the identification of patients with AD pathology.^45^ A rapidly progressive language disorder with all the initial characteristics of PPA has been described in conjunction with Jacob-Creutzfeldt disease. However, the course tends to be more rapid than in the usual cases.^46^

**Figure 1:**
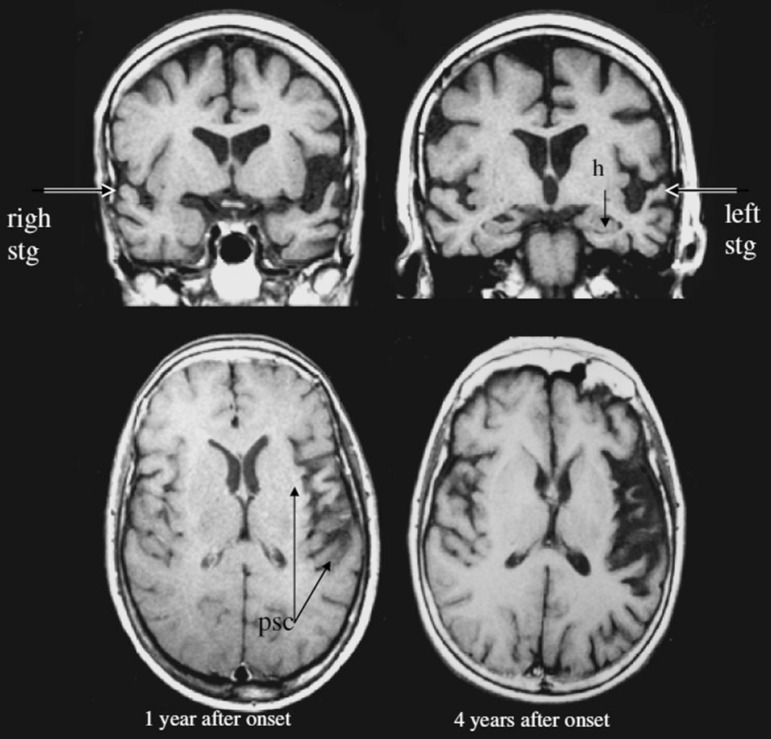
[Top] Two coronal sections, showing the asymmetric atrophy of the left perisylvian cortex in a PPA patient. [Bottom] Two axial sections showing the progression of atrophy.

## GENETICS AND RISK FACTORS OF PPA

The vast majority of PPA is sporadic. However, PPA has also been reported in dominantly inherited forms of FTLD caused by mutations in genes that encode tau (*MAPT*), progranulin (*GRN*) or the Chromosome 9 open reading frame 72 protein (*C9orf72*).^47-49^ In the group of dominantly inherited FTLD kindreds, the PPA phenotype has been described most frequently in families with *GRN* mutations. In two families, *GRN* mutations consistently resulted in the PPA phenotype.^50^ In the PPA^1^ family, three of four siblings had PPA. The mutation consisted of a single nucleotide deletion in exon 9. In the PPA3 family, two of three siblings had PPA. The mutation was a C>T transition in exon 11. Both mutations resulted in a premature termination codon and a haploinsufficiency of progranulin. The neuropathological examination in affected members of both families showed FTLD pathology with inclusions containing TDP-43. In one member of the PPA3 family unbiased stereology showed that the number of TDP-43 inclusions was higher in neocortex than in memory-related mediotemporal limbic areas, and higher in language-related neocortices of the left hemisphere than in contralateral areas on the right.^51^ The distribution of inclusions was thus concordant with the PPA profile of impaired language and relatively spared memory function.

The fit between the clinical picture and the distribution of lesions may give the impression that progranulin deficiency and the resultant TDP-43 abnormalities selectively target components of the language network. However, it is also well known that similar mutations can cause entirely different phenotypes in other families. Even within single families with *GRN* mutations, some members may have PPA, others bvFTD. The fact that identical neuropathological entities can cause PPA in some patients while causing bvFTD or amnestic dementias in others justifies the search for patient-specific susceptibility factors that interact with the neurodegenerative disease by determining its primary anatomical location.^52^

An interesting clue has emerged from an analysis of learning disabilities. We reported that learning disabilities, including dyslexia, were overrepresented in patients with PPA and their first degree relatives when compared to controls and AD patients.^9,53^ In some of these families, the concentration of dyslexia was striking, affecting the majority of children or siblings. Furthermore, two patients with PPA onset in their 60's were found to have left hemi-craniosynostosis, a mild developmental abnormality that interferes with the normal growth of the underlying cortex. In these two patients, the left hemisphere hypoplasia was functionally compensated throughout most adulthood but appears to have provided the neural background for the emergence of PPA in the 7^th^ decade of life.^54^ These observations have led us to wonder whether PPA could represent the tardive manifestation of genetic or acquired vulnerabilities of the language network that remain functionally compensated during most of adulthood but that become the locus of least resistance for the distribution of neurodegeneration. In other patients with a different set of prior vulnerabilities the same neurodegenerative process would be expected to have a different distribution and therefore different clinical manifestations.

## CONTRIBUTIONS OF PPA TO NEUROLINGUISTICS AND COGNITIVE NEUROSCIENCE

The clinicopathological correlations of classic aphasiology were based mostly on observations in patients with focal cerebrovascular lesions where the injury site, usually including cortical as well as subcortical areas, is abruptly and completely destroyed. In primary progressive aphasia, the gradual and selective loss of cortical neurons in the language network leads to more subtle perturbations and dissociations, some of which have shed new light on the neurobiology of language function. One of the most consequential new insights has been the realization that the classic neurological account of language is incomplete and that the anterior temporal lobe of the left hemisphere needs to be inserted into the language network as a third major hub with a critical role in language comprehension, especially of words denoting concrete entities.^30,55-58^ In fact, some of these observations have cast serious doubts on existing characterizations of Wernicke's area and its role in language comprehension.^59^ Another equally important insight has been the realization that grammatical ability and fluency can be dissociated neuropsychologically as well as anatomically.^60,61^ It is quite likely that future research in PPA will lead to additional revisions of the language network.

## PATIENT CARE

The manifestations of PPA are distinctly different from those of DAT. Different aspects of daily living activities are impaired and require different sorts of intervention. Some patients can learn sign language, others find it useful to carry laminated cards with specific messages, still others benefit from voice synthesizers or laptops containing digitally stored words and phrases. An evaluation by a speech therapist is useful for exploring alternative communication strategies. In contrast to DAT where new information cannot be retained in memory, the recall and evaluation of recent events remains intact although the patient may not be able to express this knowledge verbally. Explaining this phenomenon to the family and offering an objective assessment of how the aphasia interferes with verbal expression and language comprehension tends to help caregivers cope with the patient's impairments. We find that psychosocial interventions, support groups and targeted educational programs are necessary components of a comprehensive approach to patients and families.^62^

In the absence of effective treatments that can prevent, reverse or slow down the progression of AD or FTLD, there is currently no effective disease-modifying intervention for PPA. Controlled trials with bromocriptine and memantine have not yielded positive results.^63,64^ Although many patients with PPA may have atypical AD, cholinesterase inhibitors have not been particularly useful. However, a new trial of these agents, specifically in patients with the biomarkers of AD, would be useful to initiate. Anecdotal reports of success with omental transplants, intraspinal ethanercept, steroids and transcranial magnetic stimulation have appeared but need to be confirmed. A very special feature of PPA is the relative sparing of the right hemisphere for many years during the course of the disease. Stimulating the plasticity of the right hemisphere so that it can take over some of the impaired language functions remains a major and futuristic goal for treatment. Current goals in patient care include accurate clinical diagnosis of PPA at its early stages, the judicial use biomarkers to surmise the nature of the underlying neuropathology, and, whenever possible, the initiation of the most fitting multimodal interventions that address the biology of the disease as well as the specifics of the language impairment.
